# Left atrial function in young strength athletes: four-dimensional automatic quantitation study

**DOI:** 10.1007/s10554-022-02585-0

**Published:** 2022-03-07

**Authors:** Mengmeng Liu, Mengjiao Sun, Lijin Li, Pengge Li, Suyun Hou, Zhen Li, Xinxin Sun, Shaohua Hua

**Affiliations:** Department of Ultrasound, The First Affiliated Hospital of Zhengzhou University, Zhengzhou, 450000 China

**Keywords:** Four-dimensional automatic quantitation, Athlete, Left atrial function, Strain

## Abstract

Athletes might suffer from potentially fatal heart disease, which has always been a concern in cardiovascular medicine. The changes in left atrial (LA) size and function are related to the occurrence of arrhythmia. In the present study, four-dimensional automatic quantitation (4D LAQ) was used to explore the changes in LA function of young strength athletes. Eighty professional young strength athletes and sixty healthy young adults matched in age were selected for the study. The LA volumes and strains were automatically analyzed by 4D LAQ. The receiver operating characteristic (ROC) curves were used to evaluate the diagnostic value of strain in athletes' LA function. Pearson correlation analysis was performed to assess the potential association between conventional echocardiographic indexes and 4D parameters related to athletes' LA function. Compared to the control group, LA longitudinal and circumferential strain in the athlete group decreased, while LA volume increased (*P* < 0.05). However, LA strain was similar among 45 male and 35 female strength athletes (*P* > 0.05), while male athletes presented with a higher LA volume when compared to female controls (*P* < 0.05). ROC curve analysis showed that LA contraction longitudinal strain (LASct) was the best predictor in evaluating athletes' LA function. Athletes' heart rate and left ventricular mass index were significantly correlated with 4D LA function parameters.4D LAQ can be used for early detection of the changes in LA function in young strength athletes. There was no significant difference in LA strain between male and female athletes. The LASct was the most effective index for evaluating athletes' LA function.

## Introduction

High-intensity exercise training can lead to changes in hemodynamics and heart structure, which is known as “athlete's heart” [1]. With increasing reports of sudden cardiac death during and after exercise, more attention has been focused on this condition. Previous studies have shown that training could lead to significant changes not only in left ventricular (LV) structure and function, but also in the LA [2]. LA remodeling is an integral part of the “athlete's heart” that is often ignored. It has been shown to be an independent risk factor for cardiac events [3]. Our group has demonstrated a reduction in the LV global longitudinal strain in strength athletes, which was analyzed using the non-invasive pressure-strain loop area, LV remodeling, and subclinical changes in LV systolic function [4]. Some studies have shown that LA changes occur earlier than those in the LV when cardiac remodeling takes place [5]. Therefore, early detection of changes in LA structure and function is helpful to avoid or slow LV remodeling. LA strain can also be used as a reliable non-invasive index of LV filling pressure and LA dysfunction [6]. Four-dimensional automatic quantitation (4D LAQ) can determine the LA strain values in different periods in real time, and accurately evaluate the three-dimensional (3D) spatial motion of the myocardium, which is a new method to evaluate the longitudinal and circumferential LA myocardial strains [7]. The present study analyzed the changes in the LA volume and strain, and explored the value of 4D LAQ technology in evaluating LA function in athletes.

## Methods

### Study population

A total of 80 professional strength athletes participating in wrestling, boxing, judo, and weightlifting were randomly selected from the Henan heavy competitive sports management center. Of these, 45 were male and 35 were female (age range, 18–25 years). Inclusion criteria included the following: strength athletes with training experience of ≥ 5 years, those training for ≥ 30 h per week, and athletes who have never stopped training. A total of 60 young adults (30 males and 30 females) who were declared to be healthy after a physical examination, those matching in age, and athletes with similar body mass indexes (BMIs) were randomly selected as the control group. The exclusion criteria in both groups included cardiovascular disease, such as history of atrial arrhythmia, hypertrophic cardiomyopathy, and severe valvular disease, and poor image quality for myocardial speckle tracing analysis. The study was approved by the Ethics Committee of The First Affiliated Hospital of Zhengzhou University. All subjects signed the informed consent form.

### Echocardiographic analysis

Transthoracic echocardiographic examinations were performed at rest in the left lateral decubitus position with a high-quality echocardiograph (Vivid E95, GE Medical Systems, Horten, Norway) equipped with a 3.5-MHz M5S 2D and 1.5–4.0-MHz 4-V transducers. Heart rate (HR) was recorded continuously via a three-lead electrocardiogram.

### Two-dimensional and Doppler assessments

Dynamic two-dimensional grayscale images were acquired in the apical four-chamber, apical two-chamber, and LV long-axis views. Images were imported into the EchoPAC software (Version 204, GE Healthcare, Milwaukee, WI, USA) and analyzed off-line. Left atrial diameter (LAD) measurements were made in the parasternal long-axis view (2D-Mode) at end systole. LVEF was calculated by the Teichholtz method. Pulsed-wave Doppler echocardiography was used to measure early (E) and late (A) blood flow velocities through the mitral valve. The peak early diastolic velocity (e’) was measured on the septal corner of mitral annulus using tissue Doppler. The E/A and E/e’ were calculated.

### 4D volume and strain assessment

**Fig. 1:**
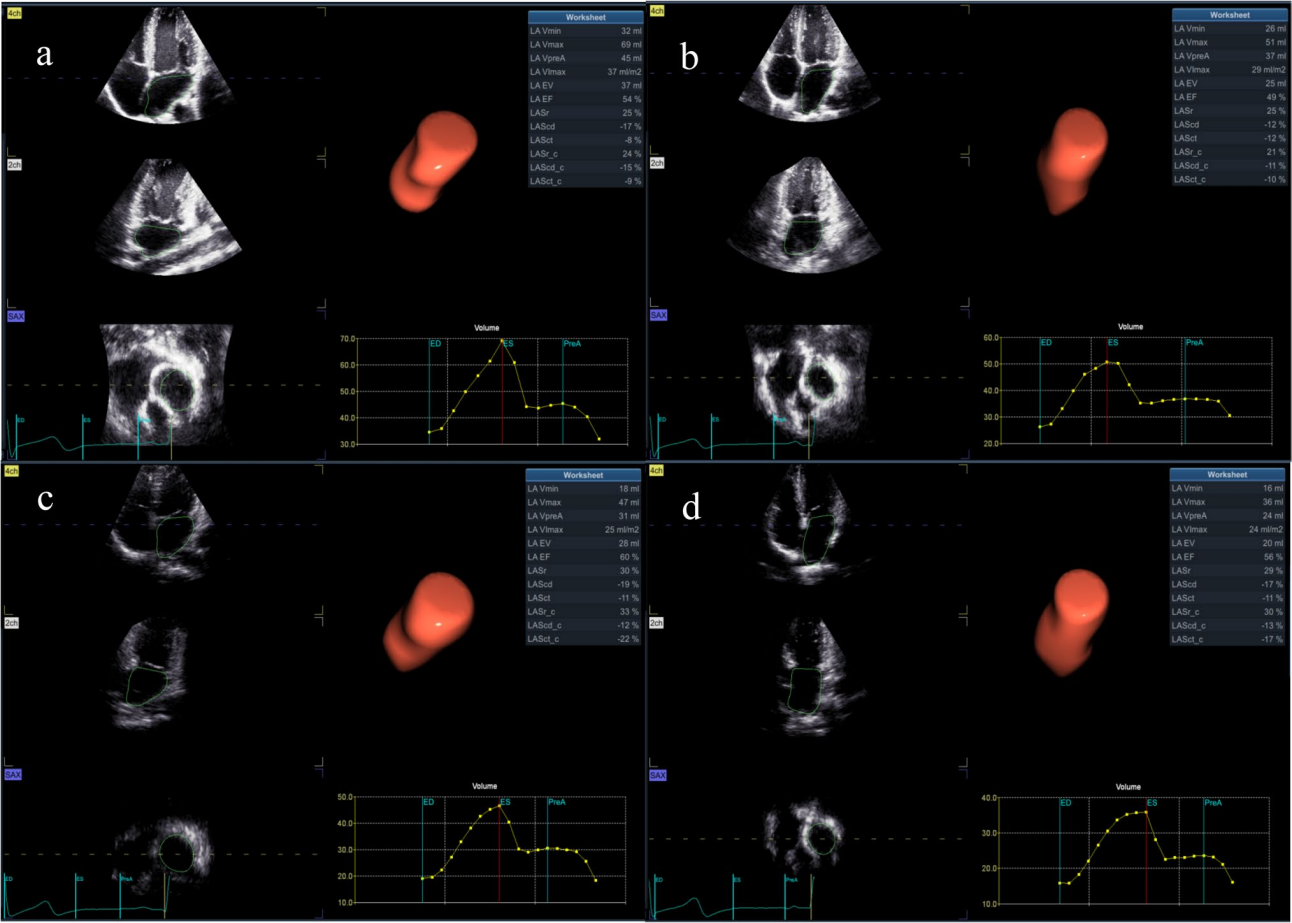
4D LA parameters by 4D LAQ **a** male athlete **b** female athlete **c** male control **d** female control

The 4D probe was used to collect the apical four-chamber full-volume dynamic images, where the volume frame rate was adjusted to be > 40% of the subjects' heart rate, and the subjects were asked to hold their breath to obtain the dynamic images. After the images were imported into the workstation, the 4D Auto LAQ option was selected and the software automatically identified and wrapped the LA intima, which could be adjusted manually to reduce the error. The 4D LA images were then obtained by selecting “Result”. The following LA parameters were analyzed by the software: maximum atrial volume (LAVmax), minimal atrial volume (LAVmin), volume before atrial contraction (LAVpre-a), left atrial ejection fraction (LAEF), left atrial reservoir longitudinal strain (LASr), conduit longitudinal strain (LAScd), contraction longitudinal strain (LASct), reservoir circumferential strain (LASr-c), conduit circumferential strain (LAScd-c), and contraction circumferential strain (LASct-c). Left atrial active ejection fraction (LAaEF) and left atrial passive ejection fraction (LApEF) were calculated using the following respective formulas: LAaEF = (LAVpre-LAVmin)/LAVpre × 100% and LApEF = (LAVmax-LAVpre)/LAVmax × 100%. The 4D LAQ technique for the analysis of the 4D LA parameters in the study subjects is shown in Fig. 1.

All ultrasound images were acquired by an experienced sonographer (a chief physician). All data were analysed by two sonographers (one attending physician and one current master's student), and the average values were taken for three consecutive measurements.

### Statistics

The analyses were performed using the statistical software, SPSS21.0 (IBM, NC, USA). Normality for the continuous variables was determined using the Kolmogorov–Smirnov test. Normally distributed continuous data with equal variance were compared between two groups using the independent samples t-test. Otherwise, the corrected t-test was used. The Mann–Whitney U test was used to compare the non-normally distributed variables in the two groups. Pearson correlation analysis was performed to assess the potential association between conventional echocardiographic indexes and 4D LA parameters. In addition, the receiver operating characteristic (ROC) curve was used to identify the optimal diagnostic indices for LA function changes. The absolute values for LAScd and LASct were used for Pearson correlation and ROC analyses.

## Results

### General data and conventional echocardiographic parameters

A total of 45 male and 35 female athletes, and 60 control subjects (30 males and 30 females) were enrolled in the present study. The demographic data and traditional echocardiography results for all participants are presented in Table 1. BSA, BMI, systolic blood pressure (SBP), LADand left atrial area (LAA) in the athlete group were higher than those in the control group, while HR and diastolic blood pressure (DBP) were lower than those in the control subjects. These differences were statistically significant (*P* < 0.05). The LV ejection fraction (LVEF), E/A, and E/e' did not differ between the two groups (*P* > 0.05). Compared to the female athlete group, BSA, BMI, SBPand LAD in the male athlete group were significantly higher and E/e' was lower (*P* < 0.05). There were no significant differences in age, HR, DBP and LVEF (*P* > 0.05).

**Table 1:** General data and conventional echocardiographic parameters

Variable	Male(75)		*P* value G1 vs G2	Female(65)		*P* value G3 vs G4	*P* value G1 vs G3	Athlete (All)	Control (All)	*P* value
	Athlete (N = 45) Group1	Control (N = 30) Group2		Athlete (N = 35) Group3	Control (N = 30) Group4					
Age (year)	20.00(3.00)	20.00(4.00)	0.150	18.00(1.00)	20.00(4.00)	0.080	0.196	18.50(3.00)	20.00(4.00)	0.096
BSA (m^2^)	2.01 ± 0.24	1.81 ± 0.09	0.000*	1.76 ± 0.15	1.58 ± 0.09	0.000*	0.000*	1.90 ± 0.23	1.69 ± 0.14	0.000*
BMI (kg/m^2^)	25.39 ± 4.72	22.31 ± 2.17	0.000*	22.49 ± 3.45	20.46 ± 1.60	0.000*	0.003*	24.09 ± 4.50	21.39 ± 2.11	0.000*
HR (bpm)	59.07 ± 8.23	72.43 ± 9.18	0.000*	56.29 ± 9.30	68.17 ± 4.03	0.000*	0.161	57.85 ± 8.77	70.30 ± 7.35	0.000*
SBP (mmHg)	132.49 ± 9.84	125.83 ± 8.20	0.003*	120.80 ± 10.85	112.80 ± 10.60	0.003*	0.000*	127.51 ± 11.88	119.32 ± 11.47	0.000*
DBP (mmHg)	74.47 ± 8.81	82.13 ± 6.45	0.000*	70.88 ± 6.35	75.03 ± 7.97	0.017*	0.084	73.11 ± 7.96	78.58 ± 8.03	0.000*
LAD (mm)	36.78 ± 2.93	33.93 ± 6.46	0.030*	35.33 ± 3.05	29.86 ± 2.10	0.000*	0.035*	36.14 ± 3.05	20.12 ± 2.74	0.000*
E/A ratio	1.86 ± 0.46	1.71 ± 0.16	0.057	2.00 ± 0.76	1.82 ± 0.39	0.217	0.326	1.92 ± 0.61	1.82 ± 0.39	0.302
E/e’ ratio	6.48 ± 1.32	6.47 ± 0.76	0.988	7.12 ± 1.63	7.02 ± 1.17	0.776	0.055	6.77 ± 1.51	7.02 ± 1.17	0.421
LVEF(%)	64.54 ± 3.28	65.66 ± 1.85	0.064	63.99 ± 2.91	64.88 ± 1.75	0.136	0.795	64.52 ± 2.98	65.17 ± 2.03	0.129

*BSA* Body surface area, *BMI* Body mass index, *HR* Heart rate, *SBP* Systolic blood pressure, *DBP* Diastolic blood pressure, *LAD* left atrial diameter; *LVEF* Left ventricular ejection fraction

*Representing significant differences (*P* < 0.05)

### 4D LA parameters

**Fig. 2:**
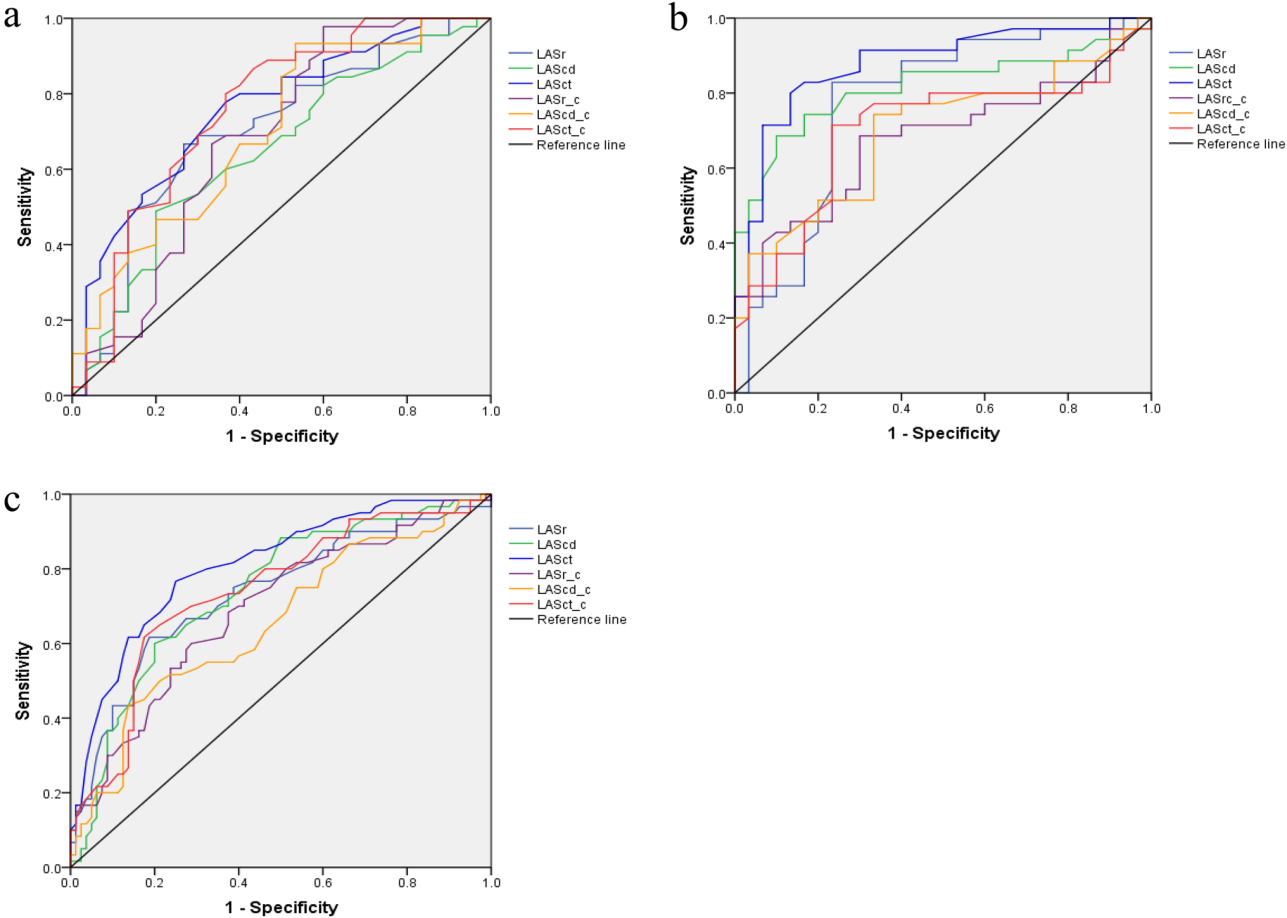
Receiver operating characteristic (ROC) curves assessed the predictive capability of left atrial strain for left atrial function. **a** male athlete vs male control **b** female athlete vs female control **c** all athlete vs all control

The comparisons of LA volumes and strains are shown in Table 2. LAVmax, LAVmin, LAVpre-a, LAVI, and LAEV were markedly larger in the athlete group than in the control group, while LAEF, LAaEF, LASr, LAScd, LASct, LASr-c, LAScd-c, and LASct-c were lower than those in the control group (*P* < 0.05). Although LApEF in the athlete group was lower than that in the control group, the difference was not statistically significant (*P* > 0.05). Compared to the female athlete group, LAVmax, LAVmin, LAVpre-a, and LAEV were higher in the male athlete group (*P* < 0.05). There were no statistical differences in LAVI, LAEF, LAaEF, LApEF, and LA strain values between the male and female athlete groups (*P* > 0.05). The LAD showed a significant inverse relationship with LA longitudinal strain and LAEF and positively correlated with LAVI. However, HR was positively correlated with LA longitudinal strain and LAEF and negatively correlated with LAVI. All differences were significant (*P* < 0.05) (Table 3). The areas under the ROC curves were 0.734, 0.741, 0.809, 0.692, 0.655, and 0.739 for predicting the ability of 4D LASr, LAScd, LASct, LASr-c, LAScd-c, and LASct-c to differentiate all athletes and controls, respectively (Fig. 2).

**Table 2:** 4D LA parameters

Variables	Male(75)		*P* value G1 vs G2	Female(65)		*P* value G3 vs G4	*P* value G1 vs G3	Athlete(All)	Control(All)	*P* value
	Athlete (N = 45) Group1	Control (N = 30) Group2		Athlete (N = 35) Group3	Control (N = 30) Group4					
LAVmin (ml)	27.63 ± 5.58	15.99 ± 4.04	0.000*	22.64 ± 6.22	16.69 ± 4.25	0.000	0.000*	25.44 ± 6.34	16.34 ± 4.13	0.000*
LAVmax (ml)	56.44 ± 12.36	35.82 ± 9.21	0.000*	45.47 ± 11.07	35.83 ± 5.93	0.000*	0.000*	51.64 ± 12.96	35.83 ± 7.68	0.000*
LAVpre-a (ml)	36.95 ± 7.82	23.67 ± 6.51	0.000*	30.19 ± 8.09	22.90 ± 5.24	0.000*	0.000*	33.99 ± 8.58	23.28 ± 5.87	0.000*
LAVI (ml/m^2^)	28.06 ± 5.89	19.83 ± 5.36	0.000*	25.71 ± 5.32	22.16 ± 3.79	0.003*	0.069	27.03 ± 5.73	20.99 ± 4.75	0.000*
LAEF (%)	50.36 ± 6.54	55.08 ± 6.68	0.003*	50.61 ± 5.66	59.03 ± 4.44	0.000*	0.856	50.47 ± 6.13	57.06 ± 5.97	0.000*
LAaEF (%)	23.95 ± 5.43	28.49 ± 4.13	0.000*	24.83 ± 5.59	29.07 ± 5.70	0.004*	0.994	24.83 ± 5.88	29.50 ± 7.39	0.000*
LApEF (%)	33.73 ± 0.40	35.06 ± 5.61	0.357	33.58 ± 6.87	36.52 ± 7.53	0.106	0.810	33.79 ± 6.71	35.22 ± 7.08	0.224
LASr (%)	21.09 ± 5.21	25.16 ± 6.25	0.003*	22.24 ± 5.40	27.88 ± 6.01	0.000*	0.341	21.59 ± 5.29	26.52 ± 6.23	0.000*
LAScd (%)	− 14.78 ± 3.68	− 16.82 ± 4.36	0.032*	− 15.54 ± 5.03	− 20.63 ± 2.79	0.000*	0.435	− 15.11 ± 4.31	− 18.73 ± 4.11	0.000*
LASct (%)	− 5.59 ± 2.87	− 8.41 ± 3.60	0.000*	− 6.58 ± 2.80	− 10.49 ± 1.87	0.000*	0.129	− 6.02 ± 2.87	− 9.45 ± 3.03	0.000*
LASr-c (%)	23.66 ± 5.38	28.81 ± 8.94	0.007*	24.79 ± 6.79	29.03 ± 5.74	0.009*	0.409	24.16 ± 6.02	28.92 ± 7.45	0.000*
LAScd-c (%)	− 13.87 ± 3.52	− 17.26 ± 4.80	0.001*	− 14.40 ± 6.02	− 18.26 ± 5.75	0.011*	0.646	− 14.10 ± 4.75	− 17.06 ± 5.89	0.001*
LASct-c (%)	− 8.68 ± 4.03	− 12.78 ± 5.71	0.000*	− 10.27 ± 3.35	− 12.32 ± 2.38	0.007*	0.064	− 9.25 ± 3.94	− 12.55 ± 4.34	0.000*

*LAVmin* minimal atrial volume, *LAVmax* maximum atrial volume, *LAVpre-a* volume before atrial contraction, *LAVI* left atrial volume index, *LAEF* left atrial ejection fraction, *LAaEF* left atrial active ejection fraction, *LApEF* left atrial passive ejection fraction, *LASr* left atrial reservioir longitudinal strain, *LAScd* conduit longitudinal strain, *LASct* contraction longitudinal strain, *LASr-c* reservioir circumferential strain, *LAScd-c* conduit circumferential strain, *LASct-c* contraction circumferential strain

*Representing significant differences (*P* < 0.05)

**Table 3:** Correlation analysis of conventional echocardiography and 4D LA parameters

Variables	LASr/%	LAScd/%	LASct/%	LAEF/%	LAVI/(ml/m^2^)
HR(bpm)	0.216*	0.211*	0.258**	0.323**	− 0.427**
LAD (mm)	− 0.337**	− 0.312**	− 0.429**	− 0.306**	0.603**
E/e’ ratio	0.149	0.178*	0.020	0.157	− 0.218**
E/A ratio	− 0.039	0.110	− 0.181*	− 0.006	0.082
LVEF(%)	0.114	0.050	0.255**	0.260**	− 0.253**

The values are Pearsons correlation coefficient

*Indicate a significant difference at the *P* = 0.05 level

**Indicate a significant difference at the *P* = 0.01 level

### Repeatability and reproducibility

The intraobserver intraclass correlation coefficients (ICCs) were 0.962, 0.985, 0.958, 0.881, 0.974, and 0.985, while the interobserver ICCs were 0.922, 0.930, 0.921, 0.872, 0.937, and 0.977 for LASr, LAScd, LASct, LASr-c, LAScd-c, and LASct-c, respectively, indicating good intra- and inter-observer agreement in the LA strain measurements.

## Discussion

It has been widely accepted that regular exercise has significant benefits, such as enhanced physical performance and metabolic homeostasis [8]. However, there is some evidence to suggest that professional athletes can be effected by adverse consequences of exercise, such as potentially lethal arrhythmias, which has been a concern for cardiovascular medicine professionals [9].

The condition known as “athlete’s heart” has different effects depending on the type of exercise. Endurance “athlete’s heart” mostly manifests as eccentric hypertrophy, while strength “athlete’s heart” is mainly characterized by concentric hypertrophy [10]. Heart remodeling in athletes occurs in both LV and LA. However, mechanisms of left atrial enlargement (LAE) are currently unknown. They are closely related to left ventricular enlargement (LVE) mainly caused by increased LV volume and pressure overload after long-term intensive training [11]. In the present study, the LAV and LA size increased significantly, whereas the ejection fraction and strain both decreased significantly in the athlete group compared to the control group. LA dilatation was more often observed in males, suggesting that LA remodeling may have already taken place. It has been theorized that LAE and remodeling may result in paroxysmal supraventricular arrhythmias [12, 13]. Animal experiments have shown that long-lasting high-intensity training led to LA remodeling and fibrosis and increased the incidence of supraventricular arrhythmias [14]. However, the correlation between the incidence of LA remodeling and supraventricular arrhythmia has not been systematically studied in high-intensity training athletes [15]. LA remodeling is also an important challenge in evaluating athletes' hearts due to the similarity of LA size between athletes and individuals with myocardial diseases, such as hypertrophic and dilated cardiomyopathies [16]. In the present study, E/e’ was used to estimate LV end-diastolic pressure, which is the best single predictor of LV filling pressure [17]. It was found that although the LA and LV in athletes had been remodeling, E/e' was normal. This showed that LA remodeling in athletes occurs in a volumetric overload model rather than in a stress overload model caused by pathological cardiac disease.

The first application of 4D LAQ to determine the LA characteristics in strength athletes and to perform early analysis of LA strain in male and female athletes represents the present study’s main strengths and innovation. The 2D-STE technique has been used to investigate the LA properties of athletes in most studies. However, it has a strong space and time dependence and easily causes speckle loss of tracking, and the analysis process is complex [18]. The 4D LAQ is a new method for assessing LA myocardial strain based on the full volumetric imaging, with the advantages of fast, comprehensive and repeatability, which can track the movement trajectory of myocardial spots in real time and quantitatively detect the motion speed and strain of LA myocardial tissue, enabling early detection of myocardial damage in athletes and providing early clinical intervention for treatment [6]. In addition, the techonology can quickly obtain the analysis results in less time and this advantage increases the clinical application of this technology. The LA morphogenesis may not be accurately reflected by 2D echocardiography due to the 3D LA structure [19]. The LAV obtained via 4D ultrasound can accurately assess the LA size. It has been suggested that LAVI obtained after LAV and corrected for BSA is more strongly correlated with adverse cardiovascular events than LAD and LAA [9, 20].

In the present study, the athlete group showed a lower LA strain and larger LAV, which helps to explain the weakening in LA myocardial deformation due to excessive atrial volume load after training. It was also found that the LAV in male athletes was greater than that in female athletes. This discrepancy is probably a result of the androgen effect on cardiac protein synthesis [21]. However, Lakatos et al. have determined that gender does not influence LA volume, which is inconsistent with our 4D data [22]. This difference may arise from the differences in race, exercise types, and training duration. A meta-analysis suggested that both LA reservoir and pump functions were lower in athletes [23]. In the present study, markers of LA reservoir function 4D LAEF, LASr, and LASr-c were significantly lower in athletes compared to those in the control subjects. LApEF reflecting LA phasic function was similar between the athlete and control groups, which might be attributed to supernormal LV early diastolic function that promotes LA emptying. Nevertheless, LAScd and LAScd-c were significantly decreased, strongly indicating that LA strain parameters are more sensitive than the volume parameters. LAaEF and LASr, which reflect LA pump function, were reduced. Studies have shown that reduced LA pump function is associated with an increased risk of atrial fibrillation [3], which may explain the higher incidence of atrial arrhythmias in elite athletes. A decrease in LA strain can be explained by the following statements: the LA wall is thin and the atrial myocardial fibers are thin and short, making them susceptible to pressure changes [24]; the intra-atrial myocardial vessels are squeezed due to prolonged exposure to atrial hypertension, leading to ischaemic degeneration and fibrous tissue proliferation of varying degrees in myocardial cells due to hypoxia [25]. The present study found a significant negative correlation between HR and LAVI, which could indicate that significant changes in HR are closely related to enlarged heart chambers. Athletes involved in high-intensity training have significantly enlarged heart chambers caused by volume overload and are able to maintain a sufficiently high cardiac output at rest even in the presence of bradycardia [26]. Engle et al. have reported a corresponding increase in LVMI in athletes with enlarged LA. The present study has found that LVMI was significantly positively correlated with LAVI and significantly negatively correlated with LA longitudinal strain, also confirming the above findings. This study also yielded the largest area under the ROC curve for LASct (AUC = 0.809), indicating that LASct is the most effective parameter in assessing the LA function in athletes.

### Limitations

Several limitations in the context of this research should be discussed. First, athletes with a family history of heart disease may not be able to provide all relevant information due to the lack of knowledge. Second, further studies with a larger sample size are needed in the future, as the present study analyzed only a small number of power athletes. Third, the reference values of 4D strain parameters in athletes obtained in the present study may differ from the actual strain reference values, and it is necessary to conduct further national multi-center studies to establish this reference range.

## Conclusion

The present study found that long-term high-intensity training can lead to LA remodeling in athletes, and 4D LAQ technology can quantitatively evaluate atrial myocardial function and detect these changes early. Since athletes can present with LAE prior to structural changes in the LV, further remodeling of the heart can be prevented from occurring early by analyzing changes in LA morphology and function. Gender had an impact on LAV rather than LA strain. LASct had the highest efficacy in evaluating LA function in athletes. LAD had a significant negative correlation with LA longitudinal strain and LAEF.

## Abbreviations

4D LAQ: Four-dimensional automatic quantitation
LA: Left atrial
ROC: Receiver operating characteristic
LV: Left ventricular
3D: Three-dimensional
BMIs: Body mass indexes
HR: Heart rate
LAD: Left atrial diameter
LAA: Left atrial area
LAVmin: Minimal atrial volume
LAVmax: Maximum atrial volume
LAVpre-a: Volume before atrial contraction
LAEF: Left atrial ejection fraction
LAaEF: Left atrial active ejection fraction
LApEF: Left atrial passive ejection fraction
LASr: Left atrial reservoir longitudinal strain
LAScd: Left atrial conduit longitudinal strain
LASct: Left atrial contraction longitudinal strain
LASr-c: Left atrial reservoir circumferential strain
LAScd-c: Left atrial conduit circumferential strain
LASct-c: Left atrial contraction circumferential strain
LVE: Left ventricular enlargement
LVEF: Left ventricular ejection fraction
